# Prediction of synergistic transcription factors by function conservation

**DOI:** 10.1186/gb-2007-8-12-r257

**Published:** 2007-12-05

**Authors:** Zihua Hu, Boyu Hu, James F Collins

**Affiliations:** 1 Center for Computational Research, New York State Center of Excellence in Bioinformatics and Life Sciences, Department of Biostatistics, Department of Medicine, University at Buffalo, State University of New York (SUNY), Buffalo, NY 14260, USA; 2 Duke University, Durham, NC 27710, USA; 3 Department of Exercise and Nutrition Sciences, University at Buffalo, State University of New York (SUNY), Buffalo, NY 14260, USA

## Abstract

**Background:**

Previous methods employed for the identification of synergistic transcription factors (TFs) are based on either TF enrichment from co-regulated genes or phylogenetic footprinting. Despite the success of these methods, both have limitations.

**Results:**

We propose a new strategy to identify synergistic TFs by function conservation. Rather than aligning the regulatory sequences from orthologous genes and then identifying conserved TF binding sites (TFBSs) in the alignment, we developed computational approaches to implement the novel strategy. These methods include combinatorial TFBS enrichment utilizing distance constraints followed by enrichment of overlapping orthologous genes from human and mouse, whose regulatory sequences contain the enriched TFBS combinations. Subsequently, integration of function conservation from both TFBS and overlapping orthologous genes was achieved by correlation analyses. These techniques have been used for genome-wide promoter analyses, which have led to the identification of 51 homotypic TF combinations; the validity of these approaches has been exemplified by both known TF-TF interactions and function coherence analyses. We further provide computational evidence that our novel methods were able to identify synergistic TFs to a much greater extent than phylogenetic footprinting.

**Conclusion:**

Function conservation based on the concordance of combinatorial TFBS enrichment along with enrichment of overlapping orthologous genes has been proven to be a successful means for the identification of synergistic TFs. This approach avoids the limitations of phylogenetic footprinting as it does not depend upon sequence alignment. It utilizes existing gene annotation data, such as those available in GO, thus providing an alternative method for functional TF discovery and annotation.

## Abstract 2

A new strategy is proposed for identifying synergistic transcription factors by function conservation, leading to the identification of 51 homotypic transcription-factor combinations.

## Background

The expression of genes is regulated by transcription factors (TFs), which interact with the basic transcription machinery to activate or repress transcription after binding to TF binding sites (TFBSs; also called *cis*-acting elements) in target genes and interacting with other DNA binding proteins. In eukaryotic organisms, transcriptional regulation of a gene's spatial, temporal, and expression level is generally mediated by multiple TFs [1-3]. Therefore, the identification of synergistic TFs and the elucidation of relationships among them are of great importance for understanding combinatorial transcriptional regulation and gene regulatory networks.

Currently, the identification of synergistic TFs comes predominantly from two general approaches. The first is by the use of experimental data such as gene expression, chromatin immunoprecipitation (ChIP)-chip, and protein-protein interaction data. For this approach, the majority of studies analyzed gene expression data across a variety of experimental conditions to infer synergistic relationships between TFs [4-9]. Statistically significant motif combinations are predicted based on stronger co-expression patterns regulated by two or more TFs than the expression patterns regulated by a single one. With the advance in protein-DNA binding assays [10], researchers have also integrated ChIP data with microarray expression or protein-protein interaction data to infer synergistic binding of cooperative TFs [11-14]. The second commonly used approach is computational identification of TF combinations. In this case, synergistic TFs were predicted by either enrichment analysis of co-occurring TFBSs on the upstream sequences of genes relative to appropriate background sequences or by comparative genomics using phylogenetically conserved sequences between closely related species [15-17].

Despite the success of these approaches, both have limitations. The approach based on experimental observation needs *a priori *knowledge, such as gene expression patterns in a certain tissue, which restricts synergistic TF determination to those tissues or cells studied and thus prevents the discovery of TF combinations from multiple biological conditions. Conversely, computational approaches can predict TF combinations on a large scale, but they usually lack the ability to functionally annotate synergistic TFs. Furthermore, methods based on phylogenetically conserved sequences, although they can greatly reduce the false prediction rate [18], have limitations related to missing potentially significant observations. Moreover, if the species are very closely related, nonfunctional sequences may not have diverged enough to allow functional sequence motifs to be identified; conversely, if the species are distantly related, short conserved regions may be masked by nonfunctional background sequences.

In the current study, rather than utilizing these traditional approaches, we propose a novel strategy to identify TF combinations by function conservation, which can be implemented at two levels. The first is functional conservation of TFs between species. Based on the strong possibility that each specific TF plays the same role in regulating gene expression between closely related species, the occurrence of its binding sites is expected to be more highly enriched in promoter sequences of orthologous genes than in promoter sequences of non-orthologous genes. The second is functional conservation of TFBSs between promoter sequences of individual orthologous genes. For identifying TF combinations, the general pattern of TFBS arrangement on promoters of orthologous genes is most likely more important than the precise positions of the binding sites [19]. To apply these concepts to synergistic TF discovery, it is important to develop appropriate computational approaches that are able to integrate function conservation from both TFs and TFBSs with analytical methodologies. We thus utilized human and mouse orthologous promoter sequences to first enrich TFBS combinations with distance constraints on a genome-scale and subsequently performed enrichment analyses of common orthologous genes (that is, genes that overlapped between mouse and human with particular homotypic TFBSs) whose regulatory sequences contain the identified TFBS combinations. We then integrated the function conservation from both levels by using Pearson correlation coefficients.

Genome-wide promoter analyses have led not only to the development of computational approaches but also to the identification of 51 homotypic TF combinations using known TFBSs from precompiled position weight matrices (PWMs) in the TRANSFAC database [20]. As a first step toward discovering functional TF networks, we have further used the developed computational approaches to predicate interactions between heterotypic TFs (that is, two different TFs). The strength of this proposed strategy, as opposed to the other described methods, lies in the fact that this strategy does not depend on sequence alignment, but rather genome information, for the discovery of functionally conserved TF combinations. Therefore, TF combinations with different functions can be obtained simultaneously, which is a key first step towards identifying functional TF networks.

## Results

### Strategy overview

**Figure 1:**
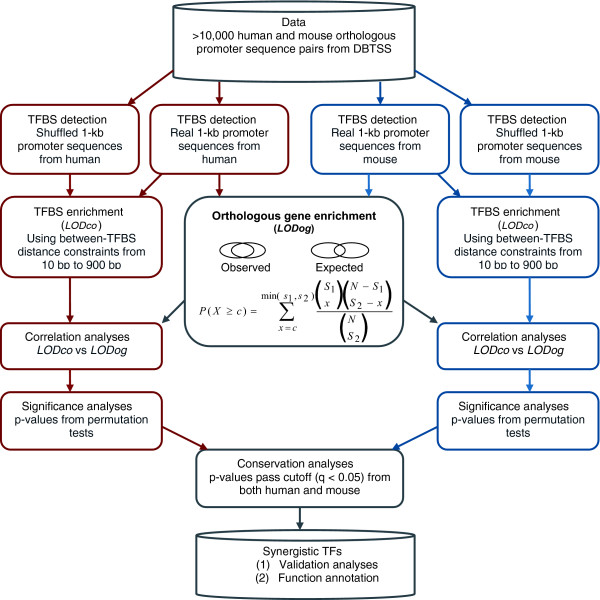
Flowchart of analysis procedures.

The overall analysis procedures are shown in Figure 1. The input data comprised more than 10,000 human and mouse orthologous promoter sequence pairs from the Database of Transcriptional Start Sites (DBTSS) [21]. To incorporate functional conservation of TFBS combinations into the analysis, we first performed a genome-wide search to obtain all potential TFBSs for each individual promoter sequence using the Match^® ^program and 234 unique PWMs from the professional TRANSFAC 9.1 database [22]. We then employed distance constraints to select co-occurring TFBSs in individual promoter sequences. The degree of enrichment for TFBS combinations was computed and represented as *LOD*_*co *_scores, which represent the frequency of co-occurrence for particular TFBSs in promoter sequences with respect to random expectation for the co-occurrence of the same TFBSs (see Materials and methods). The assumption behind this enrichment analysis is that random co-occurrence of TFBSs has less or no distance constraint when compared to functional TFBSs, although specific distance constraints may vary for different TFBSs. To incorporate functional conservation of TFs into the analysis, we estimated the degree of enrichment by using the hypergeometric distribution, which was represented as *LOD*_*og *_scores, for overlapping human and mouse orthologous genes whose promoter sequences contained the enriched TFBS combinations.

The integration of function conservation from both levels was achieved by the estimation of correlation between *LOD*_*co *_and *LOD*_*og*_. We hypothesized that if the enriched TFBS combinations had functional significance, then the enrichment of common orthologous genes would correlate with the *LOD*_*co *_scores from both human and mouse promoter sequences, since functional TFBSs are expected to be highly conserved between orthologous gene promoter sequences from closely related species. The degree of correlation would therefore allow us to identify combinatorial TFs that potentially regulated genes in a synergistic fashion. For the selection of significant correlations, we performed permutation tests to obtain *p *values, which were used to set up filtering criteria for multiple tests. Functional TF combinations were predicted based on *p *value cutoff threshold (*q*-value < 0.05) in both human and mouse and further validated by both known TF-TF interactions and function coherence based on Gene Ontology (GO) annotation of common mouse and human genes containing co-occurring TFBSs [23,24].

### Enrichment of TFBS combinations and orthologous genes containing the binding sites

For the enrichment of functionally co-occurring TFBSs, we first employed 234 PWMs, which represent unique TFs in the TRANSFAC 9.1 database, to identify homotypic TFBS combinations (that is, two or more binding sites for the same TF on the same gene). As one of the important components of the approach, a total of 18 between-TFBS distances were defined and used to obtain co-occurring TFBSs from individual promoter sequences. Enrichment of TFBS combinations was estimated on a genome-scale by comparing co-occurring TFBS frequencies in known promoter sequences to those from random background sequences.

**Figure 2:**
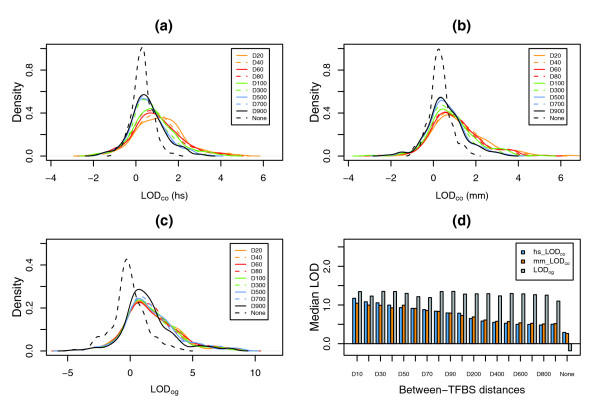
Distribution of *LOD*_*co *_and *LOD*_*og *_from different distance constraints. **(a) ***LOD*_*co *_distribution of 234 TFBSs from 9 selected distance constrains (for example, D20 stands for between-TFBS distance of 20 bp) and the one without a distance constraint (None) for human (hs). **(b) ***LOD*_*co *_distribution of 234 TFBSs from 9 selected distance constraints and the one without a distance constraint for mouse (mm). **(c) ***LOD*_*og *_distribution of 234 TFBSs from 9 selected distance constraints and the one without a distance constraint. **(d) **Median *LOD*_*co *_scores for both human (hs_LOD_co_) and mouse (mm_LOD_co_) and median *LOD*_*og *_scores.

Figure 2a,b show the overall enrichment results of TFBS combinations for 9 selected distance constraints and one without distance constraints from all 234 PWMs. A *LOD*_*co *_score > 0 exemplifies a higher frequency of TFBSs per promoter sequence when compared to background sequences. Thus, the larger the *LOD*_*co *_score, the greater the enrichment of a particular TFBS. The results show that the distributions of *LOD*_*co *_scores obtained from orthologous human and mouse promoters have similar patterns. Whereas the distribution of *LOD*_*co *_scores from the no distance constraint situation is significantly shifted in isolation to the left, *LOD*_*co *_score distributions from distance constraints are shifted to the right along with the smaller between-TFBS distances. Similar results were also obtained for enrichment of common orthologous genes containing the identified TFBS combinations, as can be seen from the *LOD*_*og *_distributions in Figure 2c.

We also performed further analyses to test the statistical significances of *LOD*_*co *_and *LOD*_*og *_score distributions from individual distance constraints using Wilcoxon signed-rank tests. The results indicated that both median *LOD*_*co *_and *LOD*_*og *_scores from individual distance constraints were significantly larger than those from no distance constraint (*p *< 10^-15^), further confirming the enrichment of co-occurring TFBSs and of common orthologous genes. It is important to note that median *LOD*_*co *_scores from individual distance constraints increase along with smaller between-TFBS distance (Figure 2d), with *p *values ranging from 2 × 10^-4 ^to 2 × 10^-16 ^for human and from 5 × 10^-8 ^to 2 × 10^-16 ^for mouse. These findings are, however, not observed for *LOD*_*og *_scores (Figure 2d), for which no significant *p *values exist from the comparisons between adjacent distance constraints. These results suggest that not all enriched TFBSs represent functional TFBS combinations and, further, that synergistic interactions may not be applicable to every homotypic TF combination.

### Integrating function conservation to identify TFs having synergistic interactions

**Figure 3:**
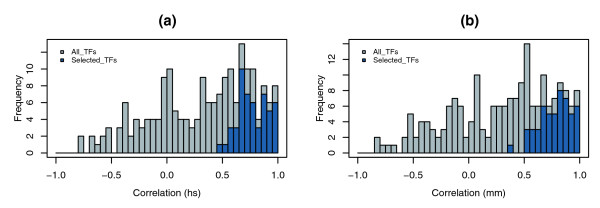
Distribution and frequency of *LOD*_*og *_and *LOD*_*co *_correlations from 19 distance constraints for individual TFBSs. **(a) **Distribution and frequency of correlation for all 243 TFBSs (grey) and for the 51 selected TFBSs (blue) from human (hs). **(b) **Distribution and frequency of correlation for all 243 TFBSs (grey) and for the 51 selected TFBSs (blue) from mouse (mm).

Since functional co-occurrence may not be applicable to every TF and not all enriched TFBSs are functional TFBS combinations, it is therefore important to integrate function conservation from different levels to predict TFs that have synergistic interactions. We employed Pearson correlation coefficients to determine whether the 19 *LOD*_*co *_scores and their corresponding *LOD*_*og *_scores for each individual TFBS correlated with each other. Since functional TFBSs are highly conserved between orthologous gene promoters, we expect that a higher rate of overlapping orthologous genes whose promoters contain the co-occurring TFBSs indicates that the enriched co-occurring TFBSs represent functional ones from individual distance constraints. Therefore, correlations detect the agreement between TFBS enrichment and orthologous gene enrichment, no matter whether all the enriched TFBSs are functional ones or not. Figure 3a,b shows the overall distributions and frequencies of correlation coefficients from all 234 TFBSs for human and mouse. While correlation coefficients cover a broad range from -0.84 to 0.99, only a small portion of TFBSs display strong correlations for their *LOD*_*co *_and *LOD*_*og *_scores, which is in agreement with the conclusion from enrichment analyses.

**Figure 4:**
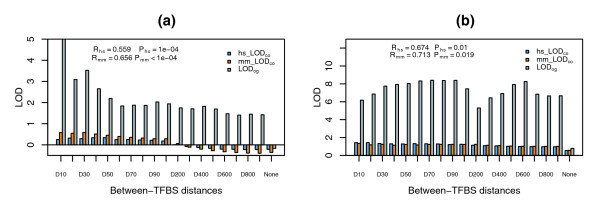
Distribution of *LOD *scores for selected TFBSs from all distance constraints. **(a) ***LOD*_*co *_scores of both human (hs_LOD_co_) and mouse (mm_LOD_co_) and *LOD*_*og *_scores for E2F1. Also shown are the correlations of *LOD*_*og *_and *LOD*_*co *_for human (*R*_*hs*_) and mouse (*R*_*mm*_) and corresponding *p *values. **(b) ***LOD*_*co *_scores of both human (hs_LOD_co_) and mouse (mm_LOD_co_) and *LOD*_*og *_scores for MYOGENIN. Also shown are the correlations of *LOD*_*og *_and *LOD*_*co *_for human (*R*_*hs*_) and mouse (*R*_*mm*_) and corresponding *p *values.

To estimate the statistical significance of the correlations, we performed permutation tests using randomly paired *LOD*_*co *_with *LOD*_*og *_scores for each TFBS and utilized the resulting *p *values to set up a cutoff threshold for multiple analyses. Using a threshold *q*-value < 0.05, we were able to identify 51 homotypic TF combinations (Table 1) from both human and mouse, with *p *values ranging from 3 × 10^-3 ^to < 10^-4 ^and correlations ranging from 0.35 to 0.98. Of these 51 TF combinations, some have relatively smaller correlations when compared to the remaining TFBSs that were not selected because they did not meet the established threshold criteria (Figure 3a,b). This is because TFBSs with similar *LOD*_*co *_and *LOD*_*og *_trends along distance constraints have smaller *p *values from permutation tests; this trend is nicely illustrated by two TFs, one that met the threshold criterion (E2F1; Figure 4a) and one that did not (MYOGENIN; Figure 4b). Although correlations for E2F1 are smaller than those for MYOGENIN, E2F1 *p *values are much more significant than those from MYOGENIN. Closer observation indicates that *LOD*_*co *_and *LOD*_*og *_scores for E2F1 show a similar trend (Figure 4a), with both increasing as between-TFBS distance decreases. By contrast, *LOD*_*co *_and *LOD*_*og *_scores for MYOGENIN do not show this trend (Figure 4b), resulting in less statistical significance, even though *LOD*_*co *_and *LOD*_*og *_scores are highly correlated. Further investigation indicated that overlapping human and mouse orthologous genes whose promoters contain predicted MYOGENIN binding site combinations had no functional association with MYOGENIN regulated genes based on GO analysis (see below), suggesting that MYOGENIN may not be a functional pair.

**Table 1:** Correlations (*R*) and *p *values (*P*) from both human (*hs*) and mouse (*mm*) for the 51 homotypic TF combinations

TFs	*R*_*hs*_	*P*_*hs*_	*R*_*mm*_	*P*_*mm*_
FAC1	0.98	<0.0001	0.98	<0.0001
MAZ	0.98	<0.0001	0.98	<0.0001
GC	0.97	<0.0001	0.99	<0.0001
ZF5	0.97	<0.0001	0.97	<0.0001
EGR	0.97	<0.0001	0.99	<0.0001
TBP	0.95	<0.0001	0.95	<0.0001
SP1	0.93	<0.0001	0.95	<0.0001
NFAT	0.93	<0.0001	0.91	<0.0001
ETF	0.92	<0.0001	0.92	<0.0001
KROX	0.90	<0.0001	0.90	<0.0001
XVENT1	0.90	<0.0001	0.86	<0.0001
ZIC3	0.90	<0.0001	0.91	<0.0001
CETS168	0.88	<0.0001	0.90	<0.0001
MZF1	0.88	<0.0001	0.89	<0.0001
PAX4	0.88	<0.0001	0.88	<0.0001
LDSPOLYA	0.87	<0.0001	0.84	<0.0001
FREAC7	0.87	<0.0001	0.84	<0.0001
OCT1	0.86	<0.0001	0.85	<0.0001
MMEF2	0.83	<0.0001	0.57	0.0007
CACBINDING PROTEIN	0.82	<0.0001	0.85	<0.0001
DEAF1	0.82	<0.0001	0.73	<0.0001
MINI19	0.78	<0.0001	0.56	0.0032
E12	0.78	0.0001	0.83	0.0001
CEBPB	0.77	<0.0001	0.80	0.0001
PU1	0.77	<0.0001	0.82	<0.0001
FOX	0.76	0.0001	0.72	<0.0001
IRF7	0.75	<0.0001	0.78	<0.0001
HNF1	0.75	0.0014	0.86	<0.0001
CETS1P54	0.74	<0.0001	0.74	<0.0001
LBP1	0.73	<0.0001	0.77	<0.0001
HNF3B	0.73	0.0006	0.67	0.0005
OSF2	0.72	0.0019	0.69	0.0005
CP2	0.71	0.0001	0.82	<0.0001
LEF1TCF1	0.70	0.0003	0.72	<0.0001
NRF2	0.68	0.0011	0.70	0.0008
TFIII	0.68	0.0005	0.65	0.0007
DBP	0.67	<0.0001	0.77	<0.0001
GATA1	0.66	0.0002	0.81	<0.0001
PIT1	0.66	<0.0001	0.67	<0.0001
HELIOSA	0.66	0.0026	0.65	0.0022
MYCMAX	0.66	0.0004	0.77	0.0001
LFA1	0.66	<0.0001	0.81	<0.0001
SRY	0.654	0.0012	0.69	0.0007
CREB	0.64	0.0003	0.55	0.0020
AP3	0.63	0.0007	0.62	0.0012
DELTAEF1	0.61	0.0016	0.52	0.0019
CAAT	0.57	0.0004	0.52	0.0030
S8	0.57	0.0004	0.64	<0.0001
E2F1	0.60	0.0001	0.67	<0.0001
NMYC	0.54	0.0005	0.58	0.0002
SRF	0.45	0.0001	0.35	0.0006

Correlations between 19 *LOD*_*co *_and their corresponding *LOD*_*og *_scores for each of 51 homotypic TF combinations are listed. Also listed are the statistical significances of the correlations computed from permutation tests using randomly paired *LOD*_*co *_with *LOD*_*og *_scores.

### Evaluation by known TF-TF interactions

To assess the validity of the predicted TF combinations, we first used TRANSCompel^® ^professional version 10.4 to determine if known TF combinations were statistically enriched in the 51 identified TFs [23]. The TRANSCompel^® ^database contains approximately 180 experimentally proven composite elements of two or more binding sites; of these approximately 180 composite elements, 15 are synergistic combinations of homotypic TFs. Interestingly, 7 of these known combinations are in the 51 selected TFs, including CEBPB, CREB, E2F1, HNF1, HNF3B, OCT1, and PIT. To estimate the degree of enrichment, we performed a Fisher's exact test comparing the occurrence of known TF combinations in the 51 identified TFs to all 234 TFs. The results indicated that known TF combinations were significantly enriched in the 51 selected TFs (*p *= 0.035) compared to those TFs that did not meet the selection criterion (*p *= 0.59). These results indicated that our approach was able to identify to a great extent functionally co-occurring TFs, which exemplifies the validity of our methods.

### Evaluation by function coherence

It is a well-established fact that TFs control cellular biological processes by targeting groups of genes encoding proteins with similar functions. Based on this fact, we performed function coherence analyses to determine if genes whose promoter sequences contained the co-occurring TFBSs had known biological functions associated with the TF predicted to bind to them. Two of the 51 selected TFs, namely E2F1 and NFAT, are of particular interest, as the genes that they regulate have well established physiological roles by previous studies. E2F1 regulates cell cycle progression via transcriptional regulation of proliferation-associated and cell cycle-related genes [25-28], while NFAT plays a central role in inducible gene transcription in the process of immune response and in the regulation of T-cell activation and differentiation [29-33].

Accordingly, we examined the functional association of genes with predicted synergistic TFBSs by looking for similar enriched GO biological process categories in overlapping human and mouse orthologous genes. This was done by first identifying the statistically over-represented GO biological process categories for genes whose promoter sequences contained the co-occurring TFBSs by DAVID [34], followed by looking for common GO biological process categories between human and mouse genes from the same distance constraint. Notably, genes whose promoter sequences contain co-occurring TFBSs display strong function coherence to the corresponding TFs binding to them, as shown in Table 2, in which enriched GO biological process categories and their *p *values from Fisher's exact tests are listed from eight distance constraints. These results indicate that identified genes with co-occurring E2F1 binding sites are involved in cell cycle control, sterol metabolism, and nucleotide and nucleic acid metabolism; notably, the biological process of cell cycle is over-represented at most distance constraints tested. In the case of NFAT, major over-represented biological functions include homophilic cell adhesion and immune response. As mentioned above, immune response is directly controlled by NFAT transcription factor. Overall, these results provide strong evidence for the functional co-occurrence of the identified TFs, and again exemplify the validity of our novel approaches.

**Table 2:** Enriched GO biological process categories for self-synergistic E2F1 and NFAT from between-TFBS distance 20 bp to 90 bp

	E2F1		NFAT	
		
Distance	No. of genes	Function categories	No. of genes	Function categories
D20	16	Cell cycle (0.07/0.09)	72	Homophilic cell adhesion (0.03/0.01)
D30	31	Sterol metabolism (0.004/0.004)	119	Homophilic cell adhesion (0.02/0.001)
				Immune response (0.06/0.003)
				Response to biotic stimulus (0.06/0.01)
				Regulation of T cell activation (0.04/0.02)
				Regulation of lymphocyte activation (0.07/0.002)
D40	49	Cell cycle (0.02/0.007)	166	Homophilic cell adhesion (0.04/0.006)
		Sterol metabolism (0.04/0.01)		Immune response (0.04/0.008)
		Nucleotide and nucleic acid metabolism (0.04/0.07)		Response to biotic stimulus (0.06/0.03)
				Regulation of T cell activation (0.07/0.03)
D50	64	Sterol metabolism (0.01/0.02)	205	Homophilic cell adhesion (0.01/0.0006)
		Cell cycle (0.005/0.02)		Immune response (0.08/0.03)
		Nucleotide and nucleic acid metabolism (0.01/0.02)		
D60	72	Cell cycle (0.002/0.009)	255	Immune response (0.03/0.002)
		Sterol metabolism (0.001/0.01)		Homophilic cell adhesion (0.03/0.002)
		Nucleotide and nucleic acid metabolism (0.008/0.02)		Response to biotic stimulus (0.09/0.01)
				Regulation of lymphocyte activation (0.06/0.02)
				Regulation of T cell activation (0.03/0.07)
				Cell-substrate adhesion (0.005/0.01)
D70	83	Cellular physiological process (0.002/0.02)	300	Homophilic cell adhesion (0.002/0.0005)
		Cell cycle (0.005/0.02)		Immune response (0.01/0.01)
		Nucleotide and nucleic acid metabolism (0.02/0.04)		Response to biotic stimulus (0.05/0.08)
		Sterol metabolism (0.002/0.003)		Regulation of lymphocyte activation (0.05/0.02)
				Cell-substrate adhesion (0.008/0.02
				Regulation of T cell activation (0.04/0.009)
D80	99	Nucleotide and nucleic acid metabolism (0.006/0.008)	341	Homophilic cell adhesion (0.0009/0.00001)
		Cell cycle (0.001/0.03)		Immune response (0.02/0.0004)
		Sterol metabolism (0.003/0.004)		Response to biotic stimulus (0.07/0.004)
		Cellular physiological process (0.001/0.01)		Regulation of lymphocyte activation (0.03/0.03)
				Cell-substrate adhesion (0.01/0.02)
D90	107	Nucleotide and nucleic acid metabolism (0.003/0.003)	392	Homophilic cell adhesion (0.0001/0.000001)
		Cell cycle (0.002/0.06)		Immune response (0.04/0.0008)
		Sterol metabolism (0.004/0.006)		Regulation of lymphocyte activation (0.05/0.04)
		Cellular physiological process (0.001/0.006)		Response to biotic stimulus (0.06/0.009)
				Cell-substrate adhesion (0.02/0.04)

The number of overlapping orthologous human and mouse genes whose promoters have at least two TF binding sites within certain distance constraints (for example, D20 for a between-TFBS distance of 20 bp) is listed under "No. of genes". The statistical significances of commonly enriched biological process categories from both human and mouse genes are listed in parentheses (*p *value mouse/*p *value human).

### Function annotation for the identified synergistic TFs

As mentioned above, it is well known that TFs control cellular biological processes via transcriptional regulation of groups of genes with similar functions. The roles of a particular TF in cellular processes can, therefore, be deduced from the known physiological functions of the TF's target genes.

**Figure 5:**
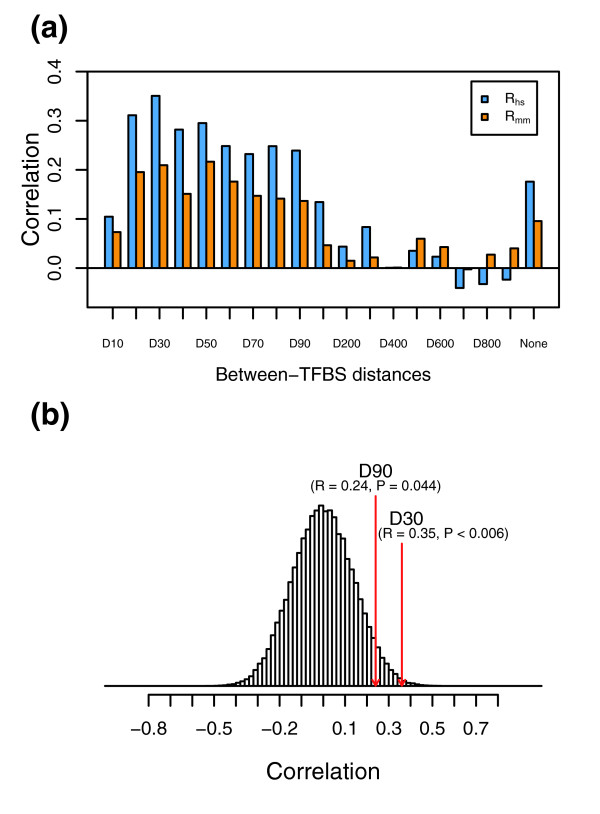
*LOD*_*og *_and *LOD*_*co *_correlation of 51 selected TFBSs from each distance constraint. **(a) **Correlation of *LOD*_*og *_and *LOD*_*co *_for all individual distance constraints for both human (*R*_*hs*_) and mouse (*R*_*mm*_). **(b) **The distribution of correlation coefficients from 100,000 permuted pairs of *LOD*_*co *_with *LOD*_*og *_scores from the between-TFBS distance of 30 bp from human. The relative locations for correlation coefficients from D30 and D90 are also shown.

To perform function annotation, while minimizing false positives, we first sought to identify distance constraints that had significant correlations between *LOD*_*co *_and *LOD*_*og *_scores from the 51 identified TFs. Accordingly, 10,000 random correlations were computed for each distance constraint using permuted *LOD*_*co *_and *LOD*_*og *_scores from the 51 TFs and used to estimate the statistical significance for real correlations. Correlations from human promoter analyses displayed significance for between-TFBS distances of 20 bp up to 90 bp, with *p *values ranging from 0.044 to 0.006 (Figure 5). Although correlations from mouse promoter analyses were not significant, 8 distance constraints (between-TFBS distances of 20 and 90 bp for human and mouse) were nevertheless used for function annotation. Common human and mouse orthologous genes containing the synergistic binding sites were subsequently submitted to DAVID for GO analysis. The selection of biological process categories for TF function annotation was based on the following criteria: biological process categories are in common in at least five distance constraints between human and mouse; there exist at least five distance constraints in both human and mouse whose *p *values for the common biological process are less than 0.05.

Function annotation results are shown in Table 3, where potential biological functions for 38 synergistic TFs are listed (significant categories were not detected from the other 13 TFs). A brief search of PubMed revealed that annotated biological functions for at least 18 of these 38 synergistic TFs are in good agreement with previously reported findings by others [35-56]. For example, earlier findings indicated that HNF1 was involved in the regulation of the expression of human organic anion transporter 3 [39], IRF in antiviral defense and immune activation [40], NRF2 in mammalian mitochondrial biogenesis [42], and Zic3 in neurogenesis [55]. All these results provide further evidence in support of our novel approaches.

**Table 3:** Function annotation for 51 homotypic TF combinations

TFs	GO biological process categories
AP3	Cell adhesion; cellular localization; cellular process; extracellular matrix organization and biogenesis; innate immune response; intracellular transport; second-messenger-mediated signaling
CAAT*	Cell cycle; cell division [36]; cell organization and biogenesis; chromosome organization and biogenesis; DNA-dependent DNA replication; nucleobase, nucleoside, nucleotide and nucleic acid metabolism; protein localization; steroid biosynthesis
CACBINDING PROTEIN	Calcium ion transport; cellular process; intracellular signaling cascade; morphogenesis; nervous system development; organ development; regulation of signal transduction
CEBPB	Cellular carbohydrate metabolism
CETSP154	Cell organization and biogenesis; cellular localization; cellular process; organelle organization and biogenesis; protein localization; ribosome biogenesis; ubiquitin cycle; vesicle-mediated transport
CETS168	Cellular physiological process
CP2	N/A
CREB	N/A
DBP	Apoptosis; cell adhesion; endocytosis; innate immune response; intracellular signaling cascade; lipid metabolism; phosphate transport; protein kinase cascade; response to endogenous stimulus; RNA processing
DEAF1	N/A
DELTAEF1	Cell adhesion
E12	N/A
E2F1*	Cell cycle [25-28]; cholesterol metabolism; nucleobase, nucleoside, nucleotide and nucleic acid metabolism; sterol metabolism
EGR*	Apoptosis; brain development; cell cycle; cell proliferation; central nervous system development; development [49]; endocytosis; enzyme linked receptor protein signaling pathway; galactose metabolism; intracellular signaling cascade; metal ion transport; nervous system development [49]; protein amino acid phosphorylation; protein kinase cascade; small GTPase mediated signal transduction; synaptic transmission; transcription [37]; ubiquitin cycle
ETF*	Cell cycle; cell proliferation; cellular lipid metabolism; central nervous system development; dephosphorylation; endocytosis; enzyme linked receptor protein signaling pathway; gluconeogenesis; heart development; hexose biosynthesis; intracellular signaling cascade; muscle development; neurite morphogenesis [38]; programmed cell death; protein kinase cascade; regulation of nucleocytoplasmic transport; response to DNA damage stimulus; small GTPase mediated signal transduction
FAC1	Cell adhesion; cell cycle; cellular lipid metabolism; endocytosis; I-kappaB kinase/NF-kappaB cascade; intracellular signaling cascade, via spliceosome; proteolysis; secretion. carbohydrate metabolism; DNA repair; nuclear import; protein kinase cascade; protein localization; response to endogenous stimulus; RNA splicing; ubiquitin cycle; vesicle-mediated transport; cytoplasm organization and biogenesis; innate immune response; endoplasmic reticulum to Golgi vesicle-mediated transport; microtubule cytoskeleton organization and biogenesis; wound healing; protein amino acid glycosylation
FOX	N/A
FREAC7	N/A
GATA1	N/A
GC*	Apoptosis [35]; cell proliferation [35]; actin cytoskeleton organization and biogenesis; cell cycle; cellular lipid metabolism; central nervous system development; endocytosis; enzyme linked receptor protein signaling pathway; endoplasmic reticulum to Golgi vesicle-mediated transport; gluconeogenesis; hexose biosynthesis; muscle development; nervous system development; Notch signaling pathway; nucleocytoplasmic transport; protein kinase cascade; small GTPase mediated signal transduction; synaptic transmission; transmembrane receptor protein tyrosine kinase signaling pathway; ubiquitin cycle; vesicle-mediated transport
HELIOSA	Cellular physiological process; development; homophilic cell adhesion; regulation of metabolism
HNF1*	Organic anion transport [39]; innate immune response
HNF3B	Lipid metabolism; DNA metabolism
IRF7*	Immune response [40]
KROX*	Actin cytoskeleton organization and biogenesis; cell cycle; enzyme linked receptor protein signaling pathway; intracellular signaling cascade; nervous system development [50]; phosphate metabolism; regulation of neurotransmitter levels; small GTPase mediated signal transduction; system development; ubiquitin cycle
LBP1*	Apoptosis [51]; cellular process; intracellular signaling cascade; protein amino acid phosphorylation; protein kinase cascade
LDSPOLYA	Development; aromatic amino acid family metabolism; intracellular signaling cascade
LEF1TDF1	N/A
LFA1	N/A
MAZ*	Apoptosis; brain development; cell adhesion; cell cycle; cell differentiation; endocytosis; enzyme linked receptor protein signaling pathway; intracellular signaling cascade; muscle development [41]; nervous system development; development [41]; protein amino acid phosphorylation; protein kinase cascade; regulation of actin filament length; small GTPase mediated signal transduction; Wnt receptor signaling
MINI19	N/A
MMEF2	N/A
MYCMAX	Cellular metabolism; macromolecule metabolism
MZF1*	Cell proliferation [52]; cell adhesion; cell cycle; cell differentiation; cell-cell signaling; enzyme linked receptor protein signaling pathway; hemopoiesis [52]; metal ion transport; nervous system development; neurotransmitter secretion; organ development; regulated secretory pathway; regulation of transcription, DNA-dependent; skeletal development; synaptic transmission; Wnt receptor signaling pathway
NFAT*	Immune response [29-31]; homophilic cell adhesion; organ development
NMYC	Cellular physiological process
NRF2*	Organelle organization and biogenesis [42]; cellular physiological process; protein transport
OCT1	Apoptosis; cell-cell adhesion; cellular physiological process; protein transport
OSF2	N/A
PAX4	Cell proliferation; enzyme linked receptor protein signaling pathway; gamma-aminobutyric acid signaling pathway; inflammatory response; programmed cell death; regulation of kinase activity
PIT1	Proteolysis
PU1	Cell adhesion; regulation of kinase activity; regulation of transferase activity
S8*	Development [46]
SP1*	Cell differentiation [53]; cell proliferation [53]; apoptosis [35]; cell adhesion; cell cycle; cell-cell signaling; cellular lipid metabolism; central nervous system development; endocytosis; nervous system development; neurogenesis; nucleocytoplasmic transport; organelle organization and biogenesis; phosphate metabolism; protein kinase cascade; response to endogenous stimulus; Rho protein signal transduction; small GTPase mediated signal transduction; synaptic transmission; transcription from RNA polymerase II promoter; transmembrane receptor protein tyrosine kinase signaling pathway; ubiquitin cycle; vesicle-mediated transport
SRF	N/A
SRY	Cell adhesion; cellular process; intracellular signaling cascade; mRNA processing; organic acid metabolism; response to DNA damage stimulus; RNA metabolism; RNA splicing; steroid metabolism
TBP	Protein transport; establishment of protein localization; RNA processing
TFIII*	Cell adhesion; cell differentiation; cell organization and biogenesis; chromatin modification; enzyme linked receptor protein signaling pathway; intracellular signaling cascade; nervous system development; organ development; protein kinase cascade; protein modification; transcription, DNA-dependent [54]
XVENT1	Cell cycle; cell growth; cell proliferation; cellular biosynthesis; establishment of cellular localization; inflammatory response; innate immune response; intracellular signaling cascade; lipid metabolism; mitochondrion organization and biogenesis; protein complex assembly; protein kinase cascade; response to endogenous stimulus; response to oxidative stress; RNA processing; RNA splicing; secretion; transcription from RNA polymerase II promoter
ZF5*	Actin polymerization and/or depolymerization; cell cycle; cell proliferation; cellular lipid metabolism; endocytosis; enzyme linked receptor protein signaling pathway; endoplasmic reticulum to Golgi vesicle-mediated transport; glycoprotein biosynthesis; hexose metabolism; induction of programmed cell death [47]; intracellular signaling cascade; JNK cascade; MAPKKK cascade; neurogenesis; phospholipid biosynthesis; protein amino acid glycosylation; protein kinase cascade; RNA splicing, via transesterification reactions; small GTPase mediated signal transduction; stress-activated protein kinase signaling pathway; ubiquitin cycle; vesicle-mediated transport; transcription from RNA polymerase II promoter [48]
ZIC3*	Cell adhesion; cell cycle; apoptosis; cell proliferation; cell-cell signaling; chromatin modification; cytoskeleton organization and biogenesis; development [56]; endocytosis; enzyme linked receptor protein signaling pathway; hexose metabolism; MAPKKK cascade; nervous system development; neurogenesis [55]; protein kinase cascade; small GTPase mediated signal transduction; striated muscle development; transmembrane receptor protein tyrosine kinase signaling pathway; vesicle-mediated transport

*TFs have corresponding functions proven by experiments from previous studies. N/A stands for no enriched or conserved biological function categories from this study.

### Functional conservation of TFBSs obviates problems associated with phylogenetic footprinting

Unlike phylogenetic footprinting, which searches for conserved TFBSs between individual orthologous genes by sequence alignment, our approach of enriching TFBS combinations involved first obtaining all potential TFBSs on a genome-scale and then looking for TFBS combinations based on the pattern of binding site arrangement on promoter sequences. For homotypic TF combinations, the pattern might include the number of TFBSs and relative between-TFBS distance. Utilizing this approach, conserved TFBS combinations that are located in different positions on the promoter sequences of orthologous genes can be identified, thus eliminating problems caused by low sequence similarity and sequence insertion or deletion.

**Figure 6:**
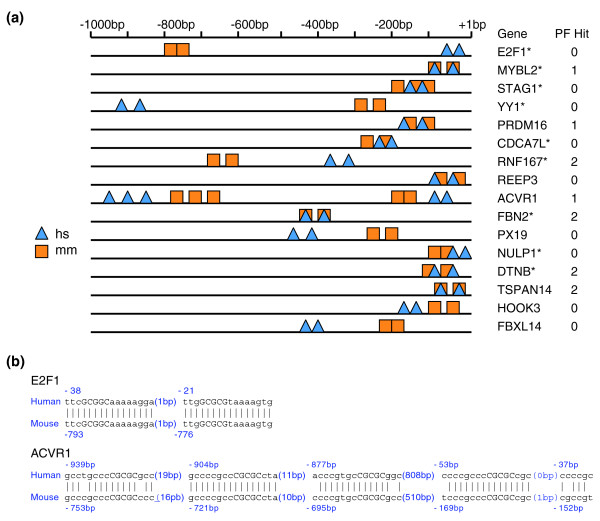
Functionally conserved E2F1 binding sites in human and mouse genes. **(a) **Schematic alignment of functionally conserved E2F1 binding sites between human (*hs*) and mouse (*mm*) promoter sequences from between-TFBS distance of 20 bp. Also listed are the numbers of conserved E2F1 binding site(s) detected by phylogenetic footprinting (PF). Asterisks indicate promoters of genes with experimentally proven E2F1 binding sites. **(b) **Sequence alignment of synergistic E2F1 binding sites from the *E2F1 *gene and two E2F1 binding site clusters from the *ACVR1 *gene. Core motifs are shown in upper case letters, and the distances between adjacent binding sites are shown in brackets. Also shown are the locations of each binding site in relation to the transcription start site.

Detailed analysis of identified E2F1 binding sites exemplifies this point; Figure 6a shows conservation of putative E2F1 binding sites on human and mouse promoter sequences from a between-TFBS distance of 20 bp. Overall, the arrangement of TFBSs is highly conserved (with the exception of one extra binding site in the mouse *STAG1 *gene). In some genes, such as the *TSPAN14 *and *FBN2 *genes, E2F1 binding sites are in exactly the same position on mouse and human promoters, while in other genes, such as the *E2F1 *and *YY1 *genes, the TFBS pairs are in vastly different locations. We thus hypothesize that it is very unlikely that traditional approaches like phylogenetic footprinting could identify all of these putative synergistic TF interactions. To test this hypothesis, we used the rVista program to perform phylogenetic footprinting to search for conserved binding sites between human and mouse promoter sequences for all genes [57]. Notably, although phylogenetic footprinting detected synergistic TFBSs for four genes, it missed the remainder (Figure 6a). It is important to note that the majority of these genes are regulated by E2F1, as their promoters were experimentally proven to be bound by E2F1. These genes include *STAG1 *[58], *YY1 *[58], *CDCA7L *[58], *RNF167 *[58], *FBN2 *[58], *NULP1 *[58], *DTNB *[58], *MYBL2 *[59], and *E2F1 *[25,60]. Phylogenetic footprinting was not able to detect any E2F1 binding site in promoters of the *E2F1*, *STAG1*, *YY1*, *CDCA7L*, and *NULP1 *genes.

To investigate whether the predicted combinatorial TFBSs that were not detected by phylogenetic footprinting are truly functional ones, we searched for genes whose promoters have experimentally proven synergistic E2F1 binding sites. One promoter of the above five genes, the E2F1 promoter, was well-characterized from both human and mouse to be synergistically bound by E2F1 (representing a self-regulatory loop) [25,60]. Sequence comparisons of both E2F1 binding sites (Figure 6b), as well as the entire promoter sequences from both human and mouse, indicated that our predicted E2F1 binding sites were exactly those experimentally proven, functional E2F1 binding site combinations on E2F1 promoters. This observation suggests that other predicted synergistic E2F1 binding sites, without experimental evidence, likely represent functionally conserved elements, despite the fact that the relative locations of binding sites might vary between species. A good example is the *ACVR1 *gene with two E2F1 binding site clusters containing a total of five putative binding sites, which show a similar arrangement between orthologous genes but are located at different positions on the promoter. A closer examination demonstrates that these two clusters are highly conserved in regards to both nucleotide sequence and spacing between each binding site within each cluster (Figure 6b), suggesting that they are indeed functionally conserved. Importantly, phylogenetic footprinting detected only one of the five E2F1 binding sites in the *ACVR1 *gene.

### Quantitative comparisons of function conservation with other methods

The above results indicated that our approach was able to identify more truly functional TFBSs than phylogenetic footprinting. We also performed further studies to make quantitative comparisons of our function conservation method with phylogenetic footprinting and the enhancer element locator (EEL) algorithm [61]; the latter also employs distance constraints to help identify interacting TFs. To facilitate this analysis, we obtained a set of 6,183 human genes whose promoters were experimentally proven to be bound by E2F1 within 1 kb upstream of the TSS in HeLa cells [58]. Out of these promoters, 1,591 (Additional data file 1) are in the promoter list of human genes used in this study and have at least one E2F1 binding site (PWM: E2F1_Q3_01). We first sought to obtain promoters with combinatorial E2F1 binding sites with given distance constraints. We subsequently computed the conditional probability that synergistic E2F1 binding sites are spaced in a given distance constraint, given E2F1 binding sites in these E2F1 target promoters. This conditional probability, as measured from real promoters, was then compared to those measured from promoters with shuffled sequences and used to compute the statistical significance for each individual distance constraint. We observed significance of E2F1 synergy for distance constraints from 10 bp to 600 bp (*p *values from 5 × 10^-4 ^to 6 × 10^-34 ^with *q*-value < 0.001) and obtained the corresponding promoters (Table 4).

**Table 4:** Significance of E2F1 synergy for different distance constraints and sensitivity/specificity for detecting synergistic E2F1 combinations by function conservation, phylogenetic footprinting, and EEL algorithm from experimentally proven E2F1 binding human promoters

	P(synergy/no. of TFBSs)									
									
Distance	Real sequences	Randomized sequences	*P *value	No. of genes*	PR_F_^†^	FPR_F_^‡^	PR_PF_^§^	FPR_PF_^¶^	PR_EEL_^¥^	FPR_EEL_^#^
D10	0.039	0.027	1.6E-04	48	10.4%	0.0%	2.1%	0.0%	6.3%	0.0%
D20	0.077	0.041	3.0E-17	92	8.7%	0.0%	3.3%	0.0%	3.3%	0.0%
D30	0.109	0.064	1.1E-18	125	8.8%	0.0%	2.4%	0.0%	5.6%	0.0%
D40	0.139	0.084	2.2E-21	159	11.9%	0.0%	3.1%	0.0%	5.7%	0.0%
D50	0.171	0.098	1.6E-31	192	12.5%	0.0%	3.1%	0.0%	6.8%	0.0%
D60	0.186	0.119	2.4E-24	209	12.0%	0.0%	3.3%	0.0%	8.1%	0.0%
D70	0.215	0.141	6.0E-26	244	12.7%	0.0%	2.9%	0.0%	7.8%	0.0%
D80	0.243	0.154	6.0E-34	272	14.7%	0.0%	2.9%	0.0%	8.1%	0.4%
D90	0.265	0.167	7.1E-38	293	14.3%	0.0%	3.1%	0.0%	7.8%	0.7%
D100	0.280	0.177	4.6E-40	309	14.2%	0.0%	3.2%	0.0%	8.1%	0.6%
D200	0.400	0.312	1.2E-22	419	17.7%	0.0%	2.6%	0.0%	7.6%	1.9%
D300	0.477	0.393	2.6E-19	487	18.1%	0.0%	2.5%	0.0%	7.2%	2.7%
D400	0.524	0.459	4.9E-12	527	19.7%	0.0%	2.3%	0.0%	7.4%	3.2%
D500	0.559	0.505	5.3E-15	557	20.3%	0.0%	2.2%	0.0%	7.7%	3.2%
D600	0.579	0.547	5.0E-04	575	20.2%	0.0%	2.1%	0.0%	8.0%	3.3%
D700	0.600	0.578	1.1E-02	-	-	-	-	-	-	-
D800	0.612	0.604	1.9E-01	-	-	-	-	-	-	-
D900	0.618	0.617	4.9E-01	-	-	-	-	-	-	-
None	0.618	0.619	5.2E-01	-	-	-	-	-	-	-

*The number of genes whose promoters have at least two E2F1 binding sites. ^†^PR_F _: positive rate from our function conservation approach. ^‡^FPR_F_: false positive rate from our function conservation approach. ^§^PR_PF_: positive rate from phylogenetic footprinting approach. ^¶^FPR_PF_: false positive rate from phylogenetic footprinting approach. ^¥^PR_EEL_: positive rate from EEL algorithm. ^#^FPR_EEL_: false positive rate from EEL algorithm.

Using these human gene promoters with combinatorial E2F1 binding sites (see Additional data file 2), we next assessed the sensitivity and specificity for detecting synergistic E2F1 combinations by function conservation and phylogenetic footprinting. In this analysis, real promoters were used as true positives and the corresponding randomized promoters with shuffled nucleotides as true negatives. Sensitivity (the fraction of promoters that were identified to have combinatorial E2F1 binding sites) was defined as the proportion of true positives over combined true positives and false negatives, and specificity as the proportion of true negatives over combined true negatives and false positives, the latter being the fraction of randomized promoters that were identified to have synergistic E2F1 combinations. We applied our function conservation, phylogenetic footprinting (using rVista), and EEL (stand-alone version for pairwise analysis) to the selected human and their corresponding mouse orthologous gene promoters. Results indicated that our function conservation approach had much higher sensitivity (approximately ten-fold) than phylogenetic footprinting for all distance constraints tested, as shown in Table 4. On the other hand, both approaches had equally excellent specificity with no false positives detected using three sets of shuffled promoter sequences. We were also curious to know the sensitivity of detecting promoters with any number of conserved E2F1 binding sites by phylogenetic footprinting. Notably, although phylogenetic footprinting was able to detect 12.9% of the 575 E2F1 target human promoters with one or more E2F1 binding sites, the positive rate was still much lower than those from our function conservation approach (20.3%) for combinatorial TFBS detection.

Results of this analysis further indicated that the EEL algorithm was able to detect conserved pairs or clusters of E2F1 sites in only 9 of the 575 target human promoters, demonstrating a much lower sensitivity (1.6%) than our function conservation approach. It is interesting to note that EEL detected multiple single E2F1 sites in many target human promoters. Although these E2F1 sites may not be conserved ones based on the underlying premise of the EEL algorithm, we nonetheless manually calculated all possible combinations of E2F1 sites for each target promoter. The overestimated positive rates are listed in Table 4, where EEL still displays much lower sensitivity (approximately 0.5-fold) than our function conservation approach for all distance constraints tested. Furthermore, false positives were detected by the EEL algorithm using three sets of shuffled promoter sequences (Table 4), indicating lower specificity for EEL. Taken together, these results indicated that our approach was able to identify conserved TFBS combinations to a much greater extent than phylogenetic footprinting and the EEL algorithm.

### Prediction of heterotypic TF interactions and TF-TF interaction networks

In an effort to expand our analyses to a more complex, perhaps more physiologically relevant situation, we applied our novel approaches to identify potential heterotypic TF combinations using the selected 51 TFs; a total of 1,275 TF combinations was considered. Correlations between *LOD*_*co *_and *LOD*_*og *_scores for these TF combinations had similar distributions to those from homotypic TF combinations, ranging from -0.96 to 0.99 for human and from -0.94 to 0.99 for mouse (data not shown). Statistical significance of the correlations estimated from permutation tests indicated that the number of significant TF combinations was overwhelming, even when a highly stringent cutoff threshold was applied (*q*-value < 0.01). Nevertheless, we selected 78 heterotypic TF combinations that passed the cutoff threshold and also had high correlations in both human and mouse (*R *> 0.85) for further TF interaction analysis. We felt that this combination of using a low *q*-value along with a high correlation coefficient would allow us to minimize false positive predictions.

**Figure 7:**
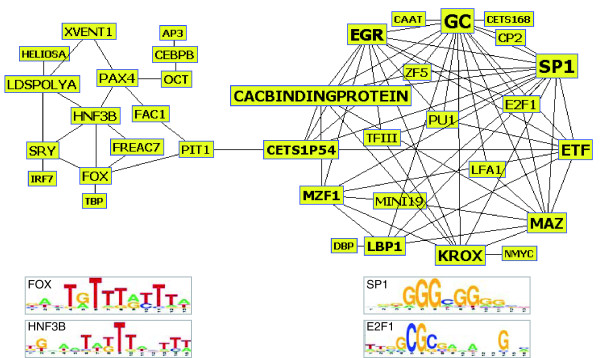
Topology of TF-TF interaction network. TF-TF relationships based on 78 synergistic TF combinations from different PWMs. Also shown are representative motif logos from both the small and the large clusters.

As shown in Figure 7, TF-TF interaction networks, utilizing these 78 heterotypic TF pairs constructed by the InterViewer program [62], segregated into two clusters, with a single link between PIT1 and CETS1P54. Thirty-six TFs were predicted to have significant interactions with one another and are represented in the figure. The smaller cluster consists of 19 synergistic TF pairs from 15 TFs, while the remaining 58 synergistic TF pairs constitute the larger cluster from 21 individual TFs. It is important to note that the binding sites for the TFs in the smaller cluster are almost all AT-enriched, while conversely, those in the larger cluster are mostly GC-enriched. A closer look at the smaller cluster reveals that HNF3B, FOX and FREAC7 (also called FOXL1), all members of the forkhead box family of transcription factors ((([63], are directly coupled to each other, suggesting that these TFs from the same family may function in a synergistic fashion. We have also performed a PubMed search to determine if SP1 is known to physically interact with any of the 13 factors to which it is connected by this analysis; we chose to delve into SP1 further as it is one of the first TFs discovered and has been highly studied. We found experimental evidence that SP1 physically interacts with EGR [64], CP2 [65], MZF1 [66], MAZ [67], PU1 [68], CETS1P54 (ETS-1) [69], E2F1 [70], ETF (TEF-1) [71], and KROX [72]. While SP1 binds to the GC-box elements and ZF5 is the analogous factor of SP1 [73,74], a couple of the factors could not be identified in PubMed by the gene names listed in Figure 7, so their interactions with SP1 are unknown. Overall, these data suggest that this approach is valid for identifying heterotypic TF interactions.

## Discussion

The identification of combinatorial TFs and the elucidation of relationships among them are of great importance for understanding transcriptional regulation as well as TF networks. Previous approaches employed for the identification of functional TF combinations are based on either TFBS enrichment from co-regulated genes or phylogenetic footprinting. Although both approaches have proven to be successful, they each have limitations. To explore alternative approaches, we propose a new strategy to look for synergistic TFs by function conservation, which was implemented from functional conservation of TFs between species and corresponding TFBSs between orthologous genes.

Although prior to our study there had not been a genome-wide function-based approach for the prediction of combinatorial TFs, several previous studies employed distance constraints to help identify interacting TFs [9,16,61], including the EEL algorithm [61]. A side-by-side comparison of the function conservation approach versus EEL is included in Table 5. It is important to point out that the core approaches for computing functional conservation at both levels were based on genome-scale information of orthologous genes, from which both TFBS combinations and common orthologous genes whose regulatory sequences contain the identified TFBS combinations were enriched. Integration of function conservation from both levels by correlation analysis led to the final prediction of combinatorial TFs. While GO analysis could provide functional annotation for the predicted TF combinations, it was employed mainly as part of our procedures to validate our approaches. Therefore, our validated approaches are readily applicable to other genomes for the prediction of physically interacting TFs and TF networks. This is likely the case as long as relative complete genes and promoter sequences are available for these genomes so that orthologous genes between closely related species can be determined correctly by pairwise alignment and cluster analysis.

**Table 5:** Comparison of function conservation approach with EEL algorithm

	Function conservation	EEL algorithm
TFBS detection	Finding all potential TFBSs	Finding all potential TFBSs
Distance constraint used	Yes	Yes
Alignment technique used	None	Non-direct DNA sequence alignment
Distance between TFBSs	Any	Relatively close
Number of genes compared	Identification of conserved TFBSs at multiple gene level	Identification of conserved TFBSs at single gene level
Parameters used for predicting interacting TFs	Conserved TFBS with function conservation of TFs at multiple gene (genome scale) level	Conserved TFBS with TF binding affinity at single gene level
Sensitivity*	Higher	Lower
Specificity*	Higher	Lower

*Relative comparison results between the function conservation approach and EEL algorithm from this study.

The enrichment of functional TFBSs plays an important role in the integration of function conservation from different levels by correlation analyses, as the functional conservation of TFs is based on genes whose promoter sequences contain over-represented binding sites. In this study, TFBS enrichment was achieved by first obtaining all potential TFBSs on a genome-scale, followed by searching for TFBS combinations by using distance constraints. Although this method was successful, other methods can also be used as long as they are able to identify over-represented TFBSs or reliably distinguish functional from non-functional TFBSs. Therefore, the strategy of function conservation is not limited to synergistic TF discovery, but is applicable to single TFs and even transcriptional regulatory modules.

The distinct advantage of using distance constraints for enrichment of TFBSs is that it not only provided a simple means for enriching functional TFBS combinations for a large number of genes, but it also allowed us to compute correlation with many enrichment results. The latter is especially important, as a large number of TFBS enrichments with changing *LOD*_*co *_score trends would contribute more to functional TFBS identification, when compared to a small number of TFBS enrichments identified by other methods. The validity of using distance constraints for enriching TFBS combinations was demonstrated not only by our study, in which the *LOD*_*co *_scores from no distance constraint were significantly smaller than those with distance constraints (*p *< 10^-15^), but also previous studies by other authors [9,16].

A related question is if there exist optimal distance constraints for all TFBSs, which would result in significant and optimal correlations between *LOD*_*co *_and *LOD*_*og *_scores for individual distance constraints. We reasoned that an optimal distance constraint is most likely to have not only a significantly large correlation but also a higher correlation than those from its two adjacent distance constraints. Interestingly, although *LOD*_*og *_and *LOD*_*co *_scores displayed general patterns of increasing correlations along with smaller between-TFBS distance (data not shown), none of these correlations were statistically significant (*p *> 0.3), based on random correlations from 10,000 permuted *LOD*_*co *_and *LOD*_*og *_scores from the same distance constraint. Although no optimal distance constraints were found for all TFBSs, we noticed that correlations between TFBS enrichment and common orthologous gene enrichment from those 51 selected TFBS combinations displayed significance for some, but not all, distance constraints (Figure 5). These results indicated that optimal distance constraints, if any, might vary among different TFBSs.

The incorporation of functional conservation of TFs can provide further stringency for synergistic TF discovery, since computational methods for enriching or characterizing functional TFBSs are likely to contain false predictions. In this study, the analyses of concordance of TFBS enrichment with overlapping orthologous gene enrichment are clearly appropriate for identifying functional TF combinations, as correlations detect the agreement between TFBS enrichment and orthologous gene enrichment, no matter whether all the enriched TFBSs are functional ones or not. We thus employed correlation analyses to achieve integration of function conservation from both TF and TFBS levels. Although correlation between *LOD*_*co *_and *LOD*_*og *_scores from each individual TFBS would provide a direct measurement for its functional co-occurrence, it did not necessarily reflect the degree of similar changing trends between *LOD*_*co *_and *LOD*_*og *_scores, which was especially important for the detection of functionally co-occurring TFBSs, as not all enriched TFBSs truly represent functional interactions. We also found that permutation tests for estimating the statistical significance of correlations is appropriate for not only setting up the cutoff threshold for the selection of the most statistically significant correlations from multiple analyses, but also for selecting TFBSs with similar trends between *LOD*_*co *_and *LOD*_*og *_scores.

The validity of functional conservation of TFBSs was also assessed by experiments from our previous investigations. In a study to identify common transcriptional regulatory elements in a group of interleukin-17 target genes [75], we employed a similar approach to obtain all potential TFBSs and looked for conserved TFBSs based on the pattern of TFBS arrangement on promoter sequences. TFs NF-κB and C/EBP were found to mediate combinatorial regulation for interleukin-17 target genes, which was validated by both luciferase reporter gene and gel shift assays. These observations provide direct proof that our computational methods can predict functional TFBSs.

In another investigation, we utilized a similar approach to identify over-represented TFBSs in a group of genes induced in the intestine of iron-deficient rats [76]. Microarray and clustering analyses led to the identification of 228 upregulated genes during iron-deficiency across several stages of postnatal development. We pulled promoter sequences for these genes in rats, mice and humans and performed enrichment analysis for TFBSs. Our results indicated that SP1-like binding sites were significantly over-represented in our experimental genes in all three species compared to random background sequences. Our analyses also predicted that SP1or a related TF could function in a synergistic fashion with the FOX TF to regulate some of our identified genes. We then inspected the promoter sequences of genes containing the predicted binding sites for the location and sequence of the TFBSs. Strikingly, we found that the SP1 and FOX binding sites were highly conserved in many of these genes; in some cases, the sequence and location in the promoters across species was identical while in other cases, the sequence and spacing were conserved, but the relative location of the binding sites was variable. These observations again emphasize the point that traditional methods such as phylogenetic footprinting are likely to miss important, conserved elements, and further that our novel approaches are a valid alternative method that should prove useful for functional TFBS prediction.

## Conclusion

Previous methods employed for the identification of functional TF combinations are based on either TFBS enrichment from co-regulated genes or phylogenetic footprinting. In this study, we have proposed the use of function conservation for the identification of TF combinations and have developed computational approaches to implement this novel strategy. These approaches include functional TFBS enrichment based on the pattern of binding site arrangement on promoters of orthologous genes by distance constraint, enrichment of overlapping orthologous genes whose regulatory sequences contain the enriched TFBS combinations, and integration of function conservation from both TF and TFBS levels by correlation analyses. To assess the usefulness of these approaches, we have applied them to human and mouse orthologous promoter sequences. Genome-wide promoter analyses have led to the identification of 51 homotypic TF combinations, which were validated by both known TF-TF interactions and function coherence analyses. The main advantage of our strategy lies in the fact that it does not depend on sequence alignment but rather genome information, thus providing an alternative method for functional TF discovery and annotation. We have further compared our approach directly with phylogenetic footprinting and clearly demonstrated that function conservation analysis was better able to predict synergistic, functional TFBSs. Overall, this study has introduced a novel approach for the discovery of functional TFBSs, which should be applicable to any species for which genome information is available.

## Materials and methods

### Promoter sequence sources

Orthologous genes between human and mouse were obtained from the 'relational table between human and mouse mutually orthologous genes' in DBTSS, which contains approximately 12,020 orthologous gene pairs. Out of these orthologous genes, 10,046 have defined promoter sequences for both human and mouse. Alternative promoter 1 was used for all genes, as alternative promoter 1 is the most upstream promoter in the case of multiple alternative promoters for a gene in DBTSS. Promoter sequences within 1 kb upstream of the annotated TSS of the 10,046 genes for both human (Additional data file 3) and mouse (Additional data file 4) were extracted from DBTSS and used for TFBS detection with the repeat sequences unmasked.

### TFBS detection

To perform the measurement for significant co-occurrence of TFBSs, an appropriate randomly generated background model is necessary. A Perl script was written to shuffle the DNA sequences within each promoter to create background sequences that have the same nucleotide content as the original promoter sequences. These background sequences are preferable to using intergenic sequences, which usually are AT-rich or exonic sequences whose nucleotide distributions tend to be biased. The resulting two sets of shuffled sequences from human and mouse, together with the original two sets of promoter sequences, were used for TFBS detection. To provide important information for estimating statistical significance for single gene pairwise comparisons, multiple shuffled sequences as background are usually employed. In our case, however, multiple sets of shuffled promoter sequences as background are not necessary, since no direct pairwise gene comparisons were performed; instead, comparisons were performed on a genome-scale using all shuffled sequences as background. The Match^® ^program [22], for which the profile parameter was set to be 'minimize the sum of false positives and negatives', was employed to conduct searches for TFBSs using vertebrate position weight matrices from the professional TRANSFAC 9.1 database. To eliminate redundant PWMs for the same TF (prefix of the 'Identifier'), the one with largest TFBS enrichment (see next section) or with highest quality control for building the PWM was selected, which resulted in 234 PWMs (Additional data file 5) for unique factors in this study.

### Enrichment of TFBS combinations by distance constraints

The co-occurrence of TFBSs was defined as two or more binding sites in the same promoter, which can be TFBSs either from the same PWM or from two PWMs. For combinatorial TFBS enrichment, distance constraints were first applied for the selection of co-occurring TFBSs with a certain defined maximum between-TFBS distance, which is the largest number of nucleotides allowed between the end of the first TFBS and the start of the next TFBS. In the case of two PWMs, the distance constraints were applied to TFBSs from 2 different PWMs but not from an identical PWM, and the number of TFBS from each individual PWM may vary. To prevent double counting of TFBSs, two overlapping matches for the same PWM were considered as a single match and the one closer to the next co-occurring TFBS was counted. A total of 18 distances were defined, ranging from the smallest 10 bp to the largest 900 bp, for which 10 bp increments were used for those with distance constraints of less than 100 bp and 100 bp increments for those with distance constraints of more than 100 bp. The counts of co-occurring TFBSs from distance constraints with smaller between-TFBS distances were also included in those with larger between-TFBS distances. The occurrence of TFBSs without a distance constraint was also computed, but overlapping TFBSs were counted only once. All calculations were separately computed using in-house developed Perl scripts (Additional data file 6).

The log odds ratio (*LOD*) was used to measure TFBS enrichment, which is the frequency of co-occurrence for a particular TFBS in real promoter sequences with respect to random expectation for the co-occurrence of the same TFBS from one corresponding set of shuffled promoter sequences:

$$LOD_{co}=ln\left(\frac{f_{r}(\#\mathrm{TFBS}/\#\mathrm{promoters})}{f_{s}(\#\mathrm{TFBS}/\#\mathrm{promoters})}\right)$$

Where *f*_*r*_*(#TFBS/#promoters) *is the TFBS frequency per gene from real promoter sequences, and *f*_*s*_*(#TFBS/#promoters) *is the frequency from shuffled promoter sequences.

### Enrichment analyses of overlapping orthologous genes

For enrichment analyses of overlapping orthologous genes, genes whose promoter sequences contain at least two co-occurring TFBSs were obtained from both human and mouse. The *p *value for the between species enrichment of overlapping orthologous genes, which contained the same TFBS combinations from the same enforced distance constraint in both human and mouse, was computed using a hypergeometric distribution:

$$P(X\geq c)=\sum_{x=c}^{\min(s_{1},s_{2})}\frac{\left(\begin{array}{l} S_{1}\\ x \end{array}\right)\left(\begin{array}{l} N-S_{1}\\ S_{2}-x \end{array}\right)}{\left(\begin{array}{l} N\\ S_{2} \end{array}\right)}$$

Where *S*_1 _and *S*_2 _are the numbers of genes whose promoters possess the co-occurring TFBSs from either mouse or human, respectively; *N *is the total number of orthologous gene pairs used in this study (*N *= 10,046); and *c *is the number of common orthologous genes between *S*_1 _and *S*_2_. The resulting *p *value is the chance probability of observing *c *or more common orthologous genes from two sets of size *S*_1 _and *S*_2 _drawn from a set of *N *gene pairs. The enrichment analyses of overlapping orthologous genes were performed for both original and shuffled promoter sequences. The latter, as described below, was obtained for the purpose of normalization.

For computing the correlation between the TFBS enrichment utilizing distance constraints and the overlapping orthologous gene enrichment, the *p *values of overlapping orthologous gene enrichment from original promoter sequences were first (-)log transformed and then normalized with the corresponding (-)log transformed *p *values from shuffled promoter sequences. To have a similar scale as *LOD*_*co*_, the *LOD *for overlapping orthologous gene enrichment was computed as follows:

$$LOD_{og}=\ln\left(\frac{-\log(p-value\_of\_orignal\_promoter)}{-\log(p-value\_of\_shuffled\_promoter)}\right)$$

### Analyses of correlations between the TFBS enrichment and the overlapping orthologous gene enrichment

Pearson correlation coefficients were employed to estimate the correlation between the 19 *LOD*_*co *_and their corresponding *LOD*_*og *_scores for each TFBS combination, and permutation tests were employed to estimate the statistical significance of the correlation. To perform the permutation tests, the 19 *LOD*_*co *_and their corresponding *LOD*_*og *_scores from each TFBS combination were randomly paired and used for random correlation computation. The procedures were repeated 10,000 times to reach a random distribution and to give sufficient power for the estimation of *p *values. The resulting *p *values were used to set up a cutoff threshold (*q*-value < 0.05) for the selection of the most statistically significant correlation from multiple analyses.

## Abbreviations

DBTSS, Database of Transcriptional Start Sites; EEL, enhancer element locator; GO, Gene Ontology; *LOD*, log odds ratio; PWM, position weight matrices; TF, transcription factor; TFBS, transcription factor binding site.

## Authors' contributions

ZH initiated and designed the study, conceived the methodology, carried out data analysis, and wrote the manuscript. BH performed the GO analysis and helped write Perl scripts. JFC participated in data analysis and writing of the manuscript. All authors read and approved the final manuscript.

## Additional data files

The following additional data are available with the online version of this paper. Additional data file 1 contains promoter sequences of 1,591 human genes from [58] with at least one E2F1 binding site (PWM: E2F1_Q3_01). Additional data file 2 contains promoter sequences of 575 human genes from [58] with at least two E2F1 binding sites of between-TFBS distance ≤ 600 bp. Additional data file 3 contains promoter sequences within 1 kb upstream of the annotated TSS for the 10,046 human genes. Additional data file 4 contains promoter sequences within 1 kb upstream of the annotated TSS for the 10,046 mouse genes. Additional data file 5 lists the matrix ID in the TRANSFAC database for the 234 PWMs used in this study. Additional data file 6 contains PERL scripts for computing TFBS combinations by distance constraints.

## Supplementary Material

Additional data file 1 Promoter sequences of 1,591 human genes from [58] with at least one E2F1 binding site (PWM: E2F1_Q3_01). Click here for file

Additional data file 2 Promoter sequences of 575 human genes from [58] with at least two E2F1 binding sites of between-TFBS distance ≤ 600 bp. Click here for file

Additional data file 3 Promoter sequences within 1 kb upstream of the annotated TSS for the 10,046 human genes. Click here for file

Additional data file 4 Promoter sequences within 1 kb upstream of the annotated TSS for the 10,046 mouse genes. Click here for file

Additional data file 5 Matrix ID in the TRANSFAC database for the 234 PWMs used in this study. Click here for file

Additional data file 6 PERL scripts for computing TFBS combinations by distance constraints. Click here for file

## References

[B1] Kel OV, Romaschenko AG, Kel AE, Wingender E, Kolchanov NA (1995). A compilation of composite regulatory elements affecting gene transcription in vertebrates.. Nucleic Acids Res.

[B2] Halfon MS, Carmena A, Gisselbrecht S, Sackerson CM, Jimenez F, Baylies MK, Michelson AM (2000). Ras pathway specificity is determined by the integration of multiple signal-activated and tissue-restricted transcription factors.. Cell.

[B3] Garten Y, Kaplan S, Pilpel Y (2005). Extraction of transcription regulatory signals from genome-wide DNA-protein interaction data.. Nucleic Acids Res.

[B4] Pilpel Y, Sudarsanam P, Church GM (2001). Identifying regulatory networks by combinatorial analysis of promoter elements.. Nat Genet.

[B5] Chiang DY, Moses AM, Kellis M, Lander ES, Eisen MB (2003). Phylogenetically and spatially conserved word pairs associated with gene-expression changes in yeasts.. Genome Biol.

[B6] Das D, Banerjee N, Zhang MQ (2004). Interacting models of cooperative gene regulation.. Proc Natl Acad Sci USA.

[B7] Keles S, van der Laan M, Eisen MB (2002). Identification of regulatory elements using a feature selection method.. Bioinformatics.

[B8] Tsai HK, Lu HH, Li WH (2005). Statistical methods for identifying yeast cell cycle transcription factors.. Proc Natl Acad Sci USA.

[B9] Yu X, Lin J, Zack DJ, Qian J (2006). Computational analysis of tissue-specific combinatorial gene regulation: predicting interaction between transcription factors in human tissues.. Nucleic Acids Res.

[B10] Lee TI, Rinaldi NJ, Robert F, Odom DT, Bar-Joseph Z, Gerber GK, Hannett NM, Harbison CT, Thompson CM, Simon I (2002). Transcriptional regulatory networks in *Saccharomyces cerevisiae*.. Science.

[B11] Banerjee N, Zhang MQ (2003). Identifying cooperativity among transcription factors controlling the cell cycle in yeast.. Nucleic Acids Res.

[B12] Kato M, Hata N, Banerjee N, Futcher B, Zhang MQ (2004). Identifying combinatorial regulation of transcription factors and binding motifs.. Genome Biol.

[B13] Smith AD, Sumazin P, Das D, Zhang MQ (2005). Mining ChIP-chip data for transcription factor and cofactor binding sites.. Bioinformatics.

[B14] Nagamine N, Kawada Y, Sakakibara Y (2005). Identifying cooperative transcriptional regulations using protein-protein interactions.. Nucleic Acids Res.

[B15] Hannenhalli S, Levy S (2002). Predicting transcription factor synergism.. Nucleic Acids Res.

[B16] Yu X, Lin J, Masuda T, Esumi N, Zack DJ, Qian J (2006). Genome-wide prediction and characterization of interactions between transcription factors in 
*Saccharomyces cerevisiae*.. Nucleic Acids Res.

[B17] Zhu Z, Shendure J, Church GM (2005). Discovering functional transcription-factor combinations in the human cell cycle.. Genome Res.

[B18] Wasserman WW, Sandelin A (2004). Applied bioinformatics for the identification of regulatory elements.. Nat Rev Genet.

[B19] Odom DT, Dowell RD, Jacobsen ES, Gordon W, Danford TW, MacIsaac KD, Rolfe PA, Conboy CM, Gifford DK, Fraenkel E (2007). Tissue-specific transcriptional regulation has diverged significantly between human and mouse.. Nat Genet.

[B20] Matys V, Fricke E, Geffers R, Gossling E, Haubrock M, Hehl R, Hornischer K, Karas D, Kel AE, Kel-Margoulis OV (2003). TRANSFAC: transcriptional regulation, from patterns to profiles.. Nucleic Acids Res.

[B21] Yamashita R, Suzuki Y, Wakaguri H, Tsuritani K, Nakai K, Sugano S (2006). DBTSS: DataBase of Human Transcription Start Sites, progress report 2006.. Nucleic Acids Res.

[B22] Kel AE, Gossling E, Reuter I, Cheremushkin E, Kel-Margoulis OV, Wingender E (2003). MATCH: A tool for searching transcription factor binding sites in DNA sequences.. Nucleic Acids Res.

[B23] Matys V, Kel-Margoulis OV, Fricke E, Liebich I, Land S, Barre-Dirrie A, Reuter I, Chekmenev D, Krull M, Hornischer K (2006). TRANSFAC and its module TRANSCompel: transcriptional gene regulation in eukaryotes.. Nucleic Acids Res.

[B24] Ashburner M, Ball CA, Blake JA, Botstein D, Butler H, Cherry JM, Davis AP, Dolinski K, Dwight SS, Eppig JT (2000). Gene ontology: tool for the unification of biology. The Gene Ontology Consortium.. Nat Genet.

[B25] Johnson DG, Ohtani K, Nevins JR (1994). Autoregulatory control of E2F1 expression in response to positive and negative regulators of cell cycle progression.. Genes Dev.

[B26] Sala A, Nicolaides NC, Engelhard A, Bellon T, Lawe DC, Arnold A, Grana X, Giordano A, Calabretta B (1994). Correlation between E2F-1 requirement in the S phase and E2F-1 transactivation of cell cycle-related genes in human cells.. Cancer Res.

[B27] Fan J, Bertino JR (1997). Functional roles of E2F in cell cycle regulation.. Oncogene.

[B28] DeGregori J, Johnson DG (2006). Distinct and overlapping roles for E2F family members in transcription, proliferation and apoptosis.. Curr Mol Med.

[B29] Rao A (1994). NF-ATp: a transcription factor required for the co-ordinate induction of several cytokine genes.. Immunol Today.

[B30] Crabtree GR, Clipstone NA (1994). Signal transmission between the plasma membrane and nucleus of T lymphocytes.. Annu Rev Biochem.

[B31] Rao A, Luo C, Hogan PG (1997). Transcription factors of the NFAT family: regulation and function.. Annu Rev Immunol.

[B32] Masuda ES, Imamura R, Amasaki Y, Arai K, Arai N (1998). Signalling into the T-cell nucleus: NFAT regulation.. Cell Signal.

[B33] Macian F (2005). NFAT proteins: key regulators of T-cell development and function.. Nat Rev Immunol.

[B34] Dennis G, Sherman BT, Hosack DA, Yang J, Gao W, Lane HC, Lempicki RA (2003). DAVID: Database for Annotation, Visualization, and Integrated Discovery.. Genome Biol.

[B35] Kaczynski J, Cook T, Urrutia R (2003). Sp1- and Kruppel-like transcription factors.. Genome Biol.

[B36] Gronostajski RM (2000). Roles of the NFI/CTF gene family in transcription and development.. Gene.

[B37] Sukhatme VP (1990). Early transcriptional events in cell growth: the Egr family.. J Am Soc Nephrol.

[B38] Yasunami M, Suzuki K, Houtani T, Sugimoto T, Ohkubo H (1995). Molecular characterization of cDNA encoding a novel protein related to transcriptional enhancer factor-1 from neural precursor cells.. J Biol Chem.

[B39] Kikuchi R, Kusuhara H, Hattori N, Shiota K, Kim I, Gonzalez FJ, Sugiyama Y (2006). Regulation of the expression of human organic anion transporter 3 by hepatocyte nuclear factor 1alpha/beta and DNA methylation.. Mol Pharmacol.

[B40] Mamane Y, Heylbroeck C, Genin P, Algarte M, Servant MJ, LePage C, DeLuca C, Kwon H, Lin R, Hiscott J (1999). Interferon regulatory factors: the next generation.. Gene.

[B41] Bossone SA, Asselin C, Patel AJ, Marcu KB (1992). MAZ, a zinc finger protein, binds to c-MYC and C2 gene sequences regulating transcriptional initiation and termination.. Proc Natl Acad Sci USA.

[B42] Scarpulla RC (2002). Nuclear activators and coactivators in mammalian mitochondrial biogenesis.. Biochim Biophys Acta.

[B43] Wirth T, Pfisterer P, Annweiler A, Zwilling S, Konig H (1995). Molecular principles of Oct2-mediated gene activation in B cells.. Immunobiology.

[B44] Kramer PR, Krishnamurthy R, Mitchell PJ, Wray S (2000). Transcription factor activator protein-2 is required for continued luteinizing hormone-releasing hormone expression in the forebrain of developing mice.. Endocrinology.

[B45] Petersenn S, Rasch AC, Penshorn M, Beil FU, Schulte HM (2001). Genomic structure and transcriptional regulation of the human growth hormone secretagogue receptor.. Endocrinology.

[B46] Norris RA, Kern MJ (2001). Identification of domains mediating transcription activation, repression, and inhibition in the paired-related homeobox protein, Prx2 (S8).. DNA Cell Biol.

[B47] Numoto M, Yokoro K, Koshi J (1999). ZF5, which is a Kruppel-type transcriptional repressor, requires the zinc finger domain for self-association.. Biochem Biophys Res Commun.

[B48] Kaplan J, Calame K (1997). The ZiN/POZ domain of ZF5 is required for both transcriptional activation and repression.. Nucleic Acids Res.

[B49] Beckmann AM, Wilce PA (1997). Egr transcription factors in the nervous system.. Neurochem Int.

[B50] Schumacher M, Guennoun R, Mercier G, Desarnaud F, Lacor P, Benavides J, Ferzaz B, Robert F, Baulieu EE (2001). Progesterone synthesis and myelin formation in peripheral nerves.. Brain Res.

[B51] Powell CM, Rudge TL, Zhu Q, Johnson LF, Hansen U (2000). Inhibition of the mammalian transcription factor LSF induces S-phase-dependent apoptosis by downregulating thymidylate synthase expression.. EMBO J.

[B52] Gaboli M, Kotsi PA, Gurrieri C, Cattoretti G, Ronchetti S, Cordon-Cardo C, Broxmeyer HE, Hromas R, Pandolfi PP (2001). Mzf1 controls cell proliferation and tumorigenesis.. Genes Dev.

[B53] Thomas K, Wu J, Sung DY, Thompson W, Powell M, McCarrey J, Gibbs R, Walker W (2007). SP1 transcription factors in male germ cell development and differentiation.. Mol Cell Endocrinol.

[B54] Kassavetis GA, Geiduschek EP (2006). Transcription factor TFIIIB and transcription by RNA polymerase III.. Biochem Soc Trans.

[B55] Inoue T, Ota M, Mikoshiba K, Aruga J (2007). Zic2 and Zic3 synergistically control neurulation and segmentation of paraxial mesoderm in mouse embryo.. Dev Biol.

[B56] Grinberg I, Millen KJ (2005). The ZIC gene family in development and disease.. Clin Genet.

[B57] Loots GG, Ovcharenko I, Pachter L, Dubchak I, Rubin EM (2002). rVista for comparative sequence-based discovery of functional transcription factor binding sites.. Genome Res.

[B58] Bieda M, Xu X, Singer MA, Green R, Farnham PJ (2006). Unbiased location analysis of E2F1-binding sites suggests a widespread role for E2F1 in the human genome.. Genome Res.

[B59] Hanada N, Lo HW, Day CP, Pan Y, Nakajima Y, Hung MC (2006). Co-regulation of B-Myb expression by E2F1 and EGF receptor.. Mol Carcinogenesis.

[B60] Hsiao KM, McMahon SL, Farnham PJ (1994). Multiple DNA elements are required for the growth regulation of the mouse E2F1 promoter.. Genes Dev.

[B61] Hallikas O, Palin K, Sinjushina N, Rautiainen R, Partanen J, Ukkonen E, Taipale J (2006). Genome-wide prediction of mammalian enhancers based on analysis of transcription-factor binding affinity.. Cell.

[B62] Ju BH, Park B, Park JH, Han K (2003). Visualization and analysis of protein interactions.. Bioinformatics.

[B63] Tan Y, Adami G, Costa RH (2002). Maintaining HNF6 expression prevents AdHNF3beta-mediated decrease in hepatic levels of Glut-2 and glycogen.. Hepatology.

[B64] Kutoh E, Schwander J (1993). Sp1 interacts with the consensus sequence for Egr-1 gene product with a cellular factor(s) and activates the transcription through this element.. Biochem Biophys Res Commun.

[B65] Xia J, Zhou ZH, Bubien JK, Fuller CM, Markert JM, Mapstone TB, Gillespie GY, Benos DJ (2003). Molecular cloning and characterization of human acid sensing ion channel (ASIC)2 gene promoter.. Gene.

[B66] Le Mee S, Fromigue O, Marie PJ (2005). Sp1/Sp3 and the myeloid zinc finger gene MZF1 regulate the human N-cadherin promoter in osteoblasts.. Exp Cell Res.

[B67] Parks CL, Shenk T (1997). Activation of the adenovirus major late promoter by transcription factors MAZ and Sp1.. J Virol.

[B68] Lopez-Rodriguez C, Corbi AL (1997). PU.1 negatively regulates the CD11c integrin gene promoter through recognition of the major transcriptional start site.. Eur J Immunol.

[B69] Sugimoto H, Okamura K, Sugimoto S, Satou M, Hattori T, Vance DE, Izumi T (2005). Sp1 is a co-activator with Ets-1, and Net is an important repressor of the transcription of CTP:phosphocholine cytidylyltransferase alpha.. J Biol Chem.

[B70] Karlseder J, Rotheneder H, Wintersberger E (1996). Interaction of Sp1 with the growth- and cell cycle-regulated transcription factor E2F.. Mol Cell Biol.

[B71] Wang SX, Elder PK, Zheng Y, Strauch AR, Kelm RJ (2005). Cell cycle-mediated regulation of smooth muscle alpha-actin gene transcription in fibroblasts and vascular smooth muscle cells involves multiple adenovirus E1A-interacting cofactors.. J Biol Chem.

[B72] Beauchef G, Kypriotou M, Chadjichristos C, Widom RL, Poree B, Renard E, Moslemi S, Wegrowski Y, Maquart FX, Pujol JP (2005). c-Krox down-regulates the expression of UDP-glucose dehydrogenase in chondrocytes.. Biochem Biophys Res Commun.

[B73] Zhao C, Meng A (2005). Sp1-like transcription factors are regulators of embryonic development in vertebrates.. Dev Growth Differ.

[B74] Yanagidani A, Matsuoka M, Yokoro K, Tanaka H, Numoto M (2000). Identification of human autoantibodies to the transcriptional repressor ZF5.. J Autoimmunity.

[B75] Shen F, Hu Z, Goswami J, Gaffen SL (2006). Identification of common transcriptional regulatory elements in interleukin-17 target genes.. J Biol Chem.

[B76] Collins JF, Hu Z (2007). Promoter analysis of intestinal genes induced during iron-deprivation reveals enrichment of conserved SP1-like binding sites.. BMC Genomics.

